# Effectiveness of a Novel Web-Based Intervention to Enhance Therapeutic Relationships and Treatment Outcomes in Adult Individual Psychotherapy: Randomized Controlled Trial and Analysis of Predictors of Dropouts

**DOI:** 10.2196/63234

**Published:** 2024-11-27

**Authors:** Alberto Stefana, Paolo Fusar-Poli, Eduard Vieta, Eric A Youngstrom

**Affiliations:** 1 Department of Brain and Behavioral Sciences University of Pavia Pavia Italy; 2 Early Psychosis: Interventions and Clinical-detection (EPIC) Lab, Department of Psychosis Studies Institute of Psychiatry, Psychology & Neuroscience King’s College London London United Kingdom; 3 OASIS Service, South London and Maudsley NHS Foundation Trust London United Kingdom; 4 Bipolar and Depressive Disorders Unit, Hospital Clinic August Pi i Sunyer Biomedical Research Institute, Centro de Investigación Biomédica en Red de Salud Mental University of Barcelona Barcelona Spain; 5 Institute for Mental and Behavioral Health Research Nationwide Children's Hospital, Division of Child and Family Psychiatry The Ohio State University Columbus, OH United States; 6 Department of Psychology and Neuroscience, University of North Carolina at Chapel Hill Chapel Hill, NC United States; 7 Helping Give Away Psychological Science Chapel Hill, NC United States

**Keywords:** therapeutic relationship, real relationship, routine outcome monitoring, measurement-based care, patient-focused research, psychotherapy process, randomized controlled trial, RCT, psychotherapy, assessment, mental health, digital mental health, eHealth, self monitoring, outcomes research, digital health, health intervention, therapy

## Abstract

**Background:**

Routine process and outcome monitoring interventions added to psychotherapy are known to improve treatment outcomes, although they vary in format and effectiveness.

**Objective:**

This study aimed to evaluate whether a therapist-independent, internet-based routine process monitoring and feedback system could significantly reduce psychological distress and enhance the quality of the therapeutic relationship compared with a treatment-as-usual control group among individuals already engaged in individual psychotherapy.

**Methods:**

We randomized 475 participants into either the intervention group, which received access to an internet-based routine process monitoring and feedback system in addition to psychotherapy, or the control group, which received only psychotherapy. The trial lasted for 10 weeks. Follow-up assessments at 5 weeks and 10 weeks used the Clinical Outcomes in Routine Evaluation-Outcome Measure as the primary outcome, with the Working Alliance Inventory-Short Revised and the Real Relationship Inventory-Client form as secondary outcomes.

**Results:**

Per-protocol analyses (n=166) showed that psychological distress decreased in both groups, but there was no significant advantage for the intervention group. The intervention group experienced a decline in the genuineness dimension score of the real relationship, with an effect size of *d*=–0.27, compared with *d*=0.01 in the control group. In the intervention group (but not in the control group), dropouts showed significantly lower real relationship levels (*P*=.002), working alliance quality (*P*=.051), and emotional disclosure (*P*=.01) compared with those who completed the study. Additionally, logistic regression revealed distinct predictors of dropout within the control group and intervention group.

**Conclusions:**

The findings do not provide conclusive evidence for the efficacy of the new internet-based intervention in enhancing self-monitoring and prompting reflection on patients’ emotional responses to their therapists. However, the intervention appears to influence patients’ perceptions of the genuineness dimension in the therapeutic relationship, warranting further investigation. We hypothesize that this alteration in the genuineness dimension could be attributed to the intervention facilitating a more realistic and accurate perception of the therapeutic relationship among participants.

**Trial Registration:**

ClinicalTrials.gov NCT06038747; https://clinicaltrials.gov/study/NCT06038747

**International Registered Report Identifier (IRRID):**

RR2-10.2196/55369

## Introduction

Psychotherapy is generally understood to consist of 2 main components: specific techniques tied to particular therapeutic models and nonspecific factors common across different approaches [1,2]. Among these common factors, the therapeutic relationship has consistently been recognized as a critical element since the inception of psychotherapy [3,4]. Initially defined as the feelings and attitudes exchanged between therapist and client, this concept has evolved to encompass broader “relational elements” that contribute to the therapeutic process across various contexts [5,6].

A meta-analysis examining outcome variance in adult psychotherapy found that approximately 30% of the variance is attributed to the patient, 15% to the therapy relationship, 10% to the specific treatment method, and 7% to the therapist [7]. Two models that have deepened our understanding of the therapeutic relationship are Wampold’s contextual model [8,9] and Gelso’s tripartite model [10,11]. Wampold’s contextual model, a “common factors” model, posits that psychotherapy—regardless of the type—operates through 3 pathways: (1) the real relationship, defined by mutual genuineness and perception of authenticity, which fosters an empathic and caring connection; (2) the patient’s expectations, shaped by their psychological beliefs, which are addressed in therapy through a rationale for the disorder and coping strategies, instilling hope; and (3) the specific ingredients of therapy, which create expectations and foster healthy behaviors unique to each therapeutic approach. Wampold’s model includes key common factors such as the therapeutic alliance, therapist empathy, and therapist effect. Gelso’s tripartite model further elaborates on the therapeutic relationship by identifying 3 interlocking elements: the real relationship, therapeutic alliance, and transference-countertransference. Notably, both models emphasize the importance of the real relationship between therapist and patient, as well as the therapeutic alliance, which encompasses the collaborative bond and shared goals that support the therapeutic process.

Specific elements of the therapeutic relationship, particularly the working alliance, are often central to routine process and outcome monitoring and feedback systems, whether directed solely to the therapist or shared with both therapist and patient [12,13]. These systems have been demonstrated to be a low-cost method for enhancing psychotherapy outcomes [14-16]. They typically use standardized metrics to assess broad treatment domains such as quality of life, symptoms, and functioning on a session-by-session basis. Evidence indicates that progress monitoring through these outcome measures can further improve the effectiveness of psychological therapies, resulting in decreased symptom intensity and lower attrition rates [14,17]. This enhancement includes a decrease in symptom intensity and lower attrition rates [17,18]. Additionally, these practices have been linked to shorter treatment durations and reduced costs [19-21]. However, the effectiveness of a web-based routine outcome monitoring and feedback system, which exclusively engages patients in completing and receiving feedback on therapeutic relationship process measures within outpatient psychotherapy, remains untested in a trial setting.

The purpose of this study was to investigate (1) whether patients who accept an additional web-based intervention continue to engage with it over time and (2) whether routine completion of a brief post-session battery that assesses the emotional responses of patients to their therapist improves individual psychotherapy outcomes. We hypothesized that a web-based intervention in addition to psychotherapy would be positively received by certain patients and would enhance the effectiveness of the therapeutic process. A secondary objective was to investigate the predictive value of demographic, clinical, and treatment characteristics for dropouts within both the control and intervention groups.

## Methods

### Participants

A total of 520 patients were recruited via the United States national online registry ResearchMatch between September 2023 and January 2024. Eligibility criteria included being 18 years or older, currently undergoing individual psychotherapy at a frequency of at least 2 sessions per month, and possessing sufficient proficiency in the English language. Exclusion criteria were confined to patients under legal guardianship. Potential participants were provided with a detailed online consent form about the study, with those interested giving their electronic consent. Participants received no financial incentives for their involvement in this trial. Although therapists were not directly informed of their patients’ participation by the research team, patients were encouraged to discuss their involvement in the study with their therapists before and during the trial.

### Procedures

Patients providing electronic informed consent underwent an online baseline assessment before being randomized into either the intervention or control group. After randomization, participants in the intervention group were provided with a link to complete a brief postsession battery following each therapy session over the course of the 10-week trial. The link for the postsession battery remained unchanged throughout the study. Each survey included a question about the date of the next session. Additionally, participants were asked if they wanted to receive a reminder email on the day of their next therapy session. If they opted in, a reminder was sent on the day of their session. Assessments for participants in both the intervention and control groups were conducted at baseline, mid-trial (5 weeks post-baseline), and end of trial (10 weeks post-baseline). Participants received 2 reminders to complete each of the follow-up assessments. Data collection was facilitated through Qualtrics.

### Ethical Considerations

Ethical approval for the study was granted by the Institutional Review Board of the University of North Carolina at Chapel Hill (reference number 23-1067). The trial was duly registered at ClinicalTrials.gov (identifier: NCT06038747), with the trial protocol published in a separate publication [22].

### Intervention

Participants allocated to the intervention group continued their regular individual psychotherapy sessions. After each session, they were asked to complete a brief postsession battery comprising 2 scales designed to evaluate affective reactions toward their therapist: the in-Session Patient Affective Reactions Questionnaire (SPARQ) [23,24] and Rift In-Session Questionnaire (RISQ) [23]. Upon completing the battery, participants received generalized feedback, emphasizing the importance of discussing their session-related feelings and reflections with their therapist. The generalized feedback was considered an integral part of the intervention and consisted of the following sentence: “Experiencing positive or negative emotions—or even a mix of both—is not uncommon in psychotherapy. It can be hard to tell our therapist about our feelings towards them, especially the ‘negative’ ones (such as feeling shy, ashamed, rejected, scared, attacked, or put down). However, sharing and discussing these feelings with your therapist can help address concerns, build trust, explore underlying issues, and, more generally, foster a supportive and collaborative therapeutic relationship that promotes personal growth and well-being.” The battery’s foremost aim was to promote participants’ self-awareness and reflection on their interactions and the overall dynamics of the therapeutic relationship.

### Control Condition

Patients in the control group received only their regular individual psychotherapy (treatment as usual) and did not complete any postsession battery. Additionally, they did not receive any communication encouraging them to discuss their session-related emotions with their therapist.

### Postsession Battery

The postsession battery incorporated 2 distinct self-report scales: the SPARQ [23,24] and RISQ [23].

The SPARQ, consisting of 8 items, delves into the cognitive, emotional, and behavioral responses patients exhibit toward their therapists during therapy sessions. This questionnaire is divided into 2 components: the Positive Affect and Negative Affect scales. Analysis of the initial session data from the trial revealed Cronbach α values of 0.86 for the Positive Affect scale and 0.79 for the Negative Affect scale.

The RISQ, comprising 4 items, aims to assess the likelihood of disruptions occurring within the therapeutic relationship. According to the findings from this study, the RISQ exhibited a Cronbach α coefficient of 0.68.

Immediately after completing the postsession battery, written feedback encouraging participants to discuss their in-session feelings and reflections with their therapist was displayed.

### Primary Outcome

The primary outcome was the level of psychological distress at the end of the 10-week trial (T2), as evaluated using the 34-item Clinical Outcomes in Routine Evaluation–Outcome Measure (CORE-OM) [25], a reliable and valid self-report measure of change in psychotherapy [25,26]. It comprises 4 domains: subjective well-being (Cronbach α=0.78), symptoms (α=0.90), function (α=0.86), and risk (α=0.84). The Cronbach α based on data at T0 was 0.95 for the total scale. A higher score on the CORE-OM indicates more severe difficulties.

### Secondary Outcomes

The level of the real relationship between the patient and therapist from the patient's point of view was evaluated with the 8-item version of the Real Relationship Inventory-Client-short form (RRI-C-SF) [27]. The real relationship is defined as “the personal relationship between therapist and patient marked by the extent to which each is genuine with the other and perceives/experiences the other in ways that befit the other” [28]. The RRI-C-SF comprises 2 subscales: genuineness (α=0.84) and realism (α=0.82), with realism referring to the accurate perception of the other. The overall scale reliability was α=0.89. Higher scores reflect a stronger real relationship.

The quality of the working alliance between patient and therapist from the patient’s perspective was assessed with the 12-item version of the Working Alliance Inventory-Short Revised (WAI-SR) [29]. The WAI-SR consists of 3 subscales: agreement on therapy goals (α=0.91), tasks (α=0.90), and the development of an affective bond (α=0.90). The total scale showed a Cronbach α of 0.95. Higher scores indicate a more robust alliance.

### Further Measures

Baseline depressive symptoms were assessed with the Patient Health Questionnaire-9 (PHQ-9) [30], while the baseline level of anxiety was measured with the Generalized Anxiety Disorder-7 (GAD-7) [31]. In this study, these scales demonstrated Cronbach α coefficients of 0.86 and 0.88, respectively.

### Sample Size Calculation

The power calculation, assuming a power of 0.80, a 2-tailed *α* of 0.05, an effect size (*d*) of 0.50, and a 1:1 allocation ratio, determined that a sample size of 128 patients (64 per group) was necessary to detect this effect size. However, to enhance statistical power and precision while accounting for a potential 50% attrition rate among participants in the intervention group, who must complete a postsession assessment after each therapy session during the 10-week trial, we aimed to enroll 520 participants (260 per group).

### Randomization

Participants were randomly assigned to either the intervention or control group using a computer-generated list following the completion of baseline assessments. Patients were not blind to the randomization.

### Data Analyses

Analyses adhered to per-protocol and intention-to-treat principles. To identify baseline differences between responders and nonresponders, unpaired *t* tests and logistic regression models were utilized. Analyses of covariance (ANCOVA) evaluated group outcomes at mid-trial and end of trial, adjusting for baseline clinical and demographic characteristics of patients, as well as treatment characteristics. Within-subject changes in outcome measures were examined using paired sample *t* tests. Findings include means, standard deviations (for ANCOVA and between-group analyses), *t* test statistics, and effect sizes (Cohen *d*), with Cohen *d* derived from ANCOVA-estimated means, adjusted for baseline scores, and corrected pooled standard deviations for group size disparities. Predictors of dropout in both control and intervention groups were explored using logistic regression analysis (completers=1), considering demographic, clinical, and treatment characteristics, as well as treatment assignment. No imputation was performed. All statistical tests were 2-sided, with a significance level set at .05, utilizing R version 4.3.1 for data analysis.

## Results

### Study Flow and Participant Characteristics

**Figure 1:**
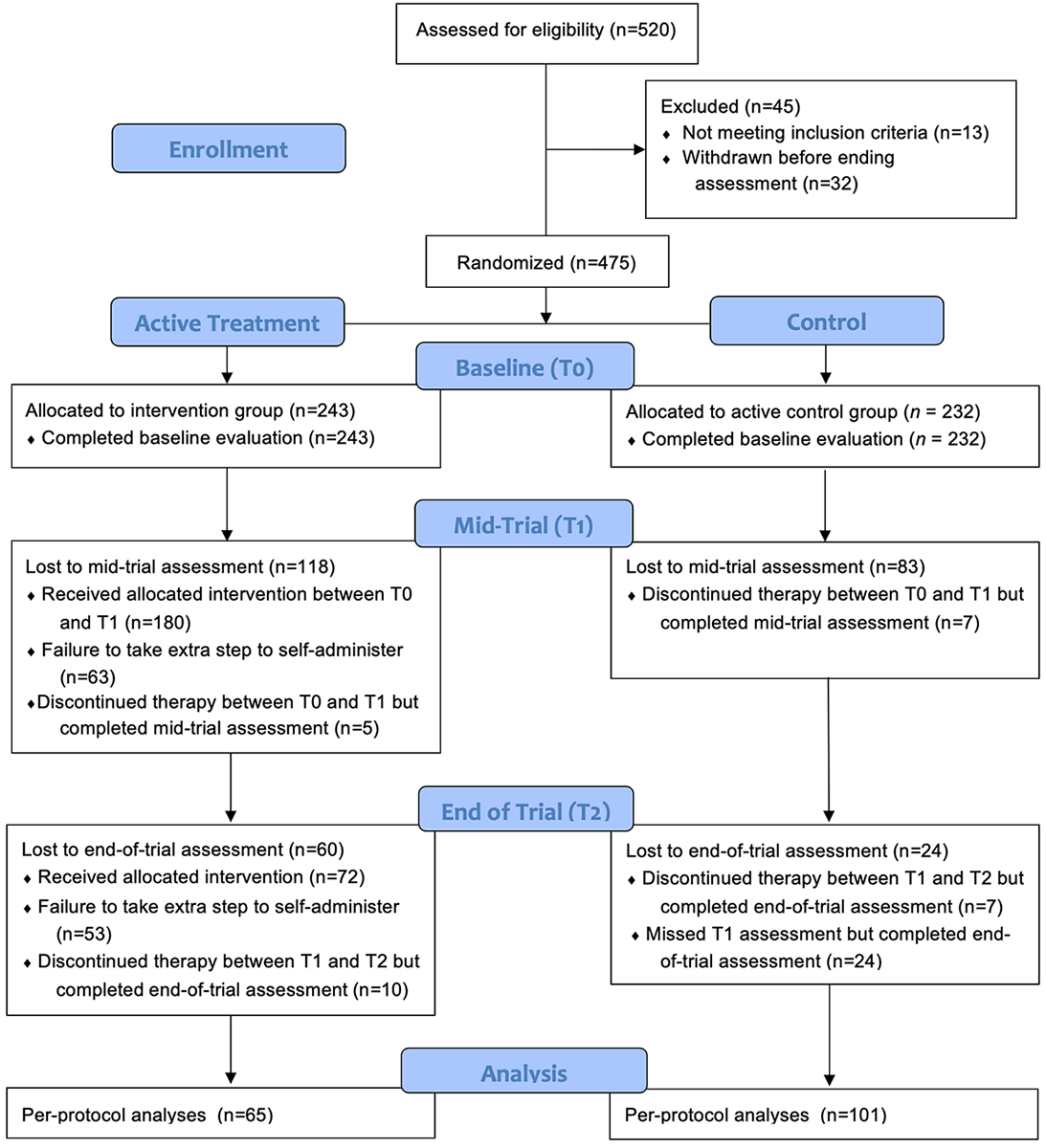
CONSORT (Consolidated Standards of Reporting Trials) flow diagram showing the flow of participants in the trial.

Figure 1 shows the flow of the study participants from recruitment to the end of the intervention (T2). Of the 520 patients recruited, 45 (8.7%) were excluded from the study for not meeting the inclusion criteria (ie, a minimum of 2 therapy sessions per month—13/45, 2.9%) or for not completing the baseline assessment (32/520, 6.2%). Thus, a total of 475 (91.3%) patients were randomized, with 243 allocated to the intervention group and 232 allocated to the control group. The discrepancy (n=11) between the number of individuals randomized to the active treatment group (n=243) and the control group (n=232) resulted from the use of a pre-established computer-generated randomization list based on a sample of 520. This list did not account for the exclusion of 45 patients who either did not meet the inclusion criteria or withdrew during the baseline assessment.

Regarding the control group, 64.2% (149/232) of the participants completed the mid-trial assessment (T1), and 53.9% (125/232) completed the end-of-trial assessment (T2). A total of 48 participants completed T1 but not T2, while 24 participants missed T1 but completed T2. Between T0 and T1, 7 patients terminated their psychotherapy but completed the mid-trial assessment, and between T1 and T2, another 7 patients terminated their psychotherapy but completed the end-of-trial assessment.

In the intervention group, 180 (180/242, 74.4%) patients completed at least 1 postsession battery and read the feedback. At T1, 51.4% (125/243) of the participants completed the assessment, and 26.7% (65/243) completed the assessments at both T1 and T2. Between T0 and T1, 5 patients terminated their psychotherapy but completed the mid-trial assessment, and between T1 and T2, 10 patients terminated their psychotherapy but completed the end-of-trial assessment.

The attrition rate at T1 was 35.8% (83/232) for the control group and 48.6% (118/243) for the intervention group, showing a statistically significant difference (*P*=.005). At T2, the attrition rate increased to 46.1% (107/232) for the control group and 73.3% (178/243) for the intervention group (*P*<.001).

Sociodemographic and clinical characteristics of both the intention-to-treat and the per-protocol samples are reported in Table 1. Participants in the intention-to-treat sample were predominantly women (361/475, 76%), were in the mean age range of 30 years to 39 years (142/475, 29.9%), and held bachelor’s degrees or higher. Most participants had a diagnosis of a psychiatric disorder (427/475, 89.9%) and received a psychotropic medication (337/475, 70.9%). Many were in psychotherapy for more than 24 months (225/475, 47.4%), typically at a frequency of 2 to 3 sessions per month (255/475, 53.7%). Participants in the intervention and control groups did not differ statistically significantly for sociodemographic and clinical characteristics at baseline (all *P* values ≥.057).

Participants who completed the entire trial did not significantly differ from those who dropped out regarding sociodemographic characteristics or scores on primary and secondary outcome measures as measured at baseline (before randomization). However, significant differences were observed in some clinical features and treatment characteristics (see Multimedia Appendix 1). Notably, the nature of these significant differences varied between dropouts in the control group and those in the intervention group. In the intervention group, participants without a diagnosed psychiatric disorder were more likely to drop out (*P*=.002), as were those with lower baseline levels of real relationship as measured by the RRI-C-SF (*P*=.002), those with a lower tendency to disclose their emotional reactions to the therapist (*P*=.01), those attending fewer sessions per month (*P*=.02), and those who canceled more sessions during the 5 weeks preceding the trial (*P*=.005). Similarly, among control participants, a lower quality of working alliance as measured by the WAI-SR was almost statistically significantly associated with a higher dropout likelihood (*P*=.051). In the intervention group, the dropout rate was higher among participants receiving treatment in public health institutions and lower among those in private health institutions (*P*=.04).

**Table 1:** Demographic, clinical, and treatment characteristics of participating patients.

Characteristics				Intention-to-treat sample						Per-protocol sample				
				Control group (n=232)		Intervention group (n=243)		*P* value		Control group (n=101)		Intervention group (n=65)		*P* value
**Sociodemographic features**														
	**Age (years), n (%)**							.34						.20
		18-22	14 (6)		16 (6.6)				8 (7.9)		2 (3.1)			
		23-29	61 (26.3)		54 (22.3)				23 (22.8)		9 (13.8)			
		30-39	74 (31.9)		68 (28)				33 (32.7)		19 (29.2)			
		40-49	30 (12.9)		46 (18.9)				12 (11.9)		15 (23.1)			
		50-59	32 (13.8)		29 (11.9)				15 (14.9)		10 (15.4)			
		≥60	21 (9.1)		30 (12.3)				10 (9.9)		10 (15.4)			
	**Gender, n (%)**							.91						.41
		Woman	176 (75.9)		185 (76.1)				73 (72.3)		54 (83.1)			
		Man	38 (16.4)		38 (15.6)				20 (19.8)		8 (12.3)			
		Other	16 (6.9)		19 (7.8)				7 (6.9)		3 (4.6)			
		Prefer not to say	2 (0.9)		1 (0.4)				1 (1)		0 (0)			
	**Education, n (%)**							.12						.26
		Less than high school	3 (1.3)		0 (0)				0 (0)		0 (0)			
		High school graduate	15 (6.5)		6 (2.5)				5 (5)		1 (1.5)			
		Some college	44 (19)		39 (16)				14 (13.9)		9 (13.8)			
		2-year degree	22 (9.5)		19 (7.8)				10 (9.9)		2 (3.1)			
		4-year degree	73 (31.5)		87 (35.8)				38 (37.6)		21 (32.3)			
		Professional degree	62 (26.7)		75 (30.9)				27 (26.7)		25 (38.5)			
		Doctorate	13 (5.6)		17 (7)				7 (6.9)		7 (10.8)			
**Clinical characteristics**														
	**Diagnosis of a mental disorder, n (%)**							.64						.03
		No	25 (10.8)		23 (9.5)				7 (6.9)		0 (0)			
		Yes	207 (89.2)		220 (90.5)				94 (93.1)		65 (100)			
	**Diagnoses^a^, n**													
		Anxiety disorders	171		185		.40		79		53		≥.99	
		Bipolar disorders	35		37		.90		11		12		.33	
		Depressive disorders	142		155		.41		68		49		.78	
		Disruptive behavior and dissocial disorders	3		4		.73		0		3		.07	
		Eating disorders	29		21		.32		14		5		.30	
		Neurodevelopmental disorders	68		65		.86		31		17		.56	
		Post-traumatic stress disorder	97		82		.26		46		28		.73	
		Psychotic disorders	5		5		≥.99		3		1		.65	
		Cluster A personality disorder	3		4		.73		1		0		≥.99	
		Cluster B personality disorder	26		19		.37		12		8		≥.99	
		Cluster C personality disorder	22		13		.17		5		5		.53	
		Other(s)	11		21		.11		5		5		≥.99	
	**Psychiatric medication use, n (%)**							.18						.45
		No	74 (31.9)		64 (26.3)				27 (26.7)		14 (21.5)			
		Yes	158 (68.1)		179 (73.7)				74 (73.3)		51 (78.5)			
	**Medication stability, n (%)**							.62						.86
		≤1 month	17 (10.8)		22 (12.3)				8 (10.8)		6 (11.8)			
		2 months	21 (13.3)		18 (10.1)				8 (10.8)		7 (13.7)			
		≥3 months	120 (75.9)		139 (77.7)				58 (78.4)		38 (74.5)			
	**Psychological measures, mean (SD)**													
		Total CORE-OM^b^ score	53.1 (24)		51.7 (24)		.501		50.5 (24.5)		47.6 (24.5)		.46	
		CORE-OM well-being score	8.0 (3.6)		8.0 (3.6)		.92		7.7 (3.7)		7.5 (4.0)		.79	
		CORE-OM symptoms score	22.5 (10.8)		22.2 (10.2)		.77		21.8 (10.8)		20.6 (10.0)		.45	
		CORE-OM functioning score	20.3 (9.1)		19.3 (9.2)		.23		19.0 (9.1)		17.7 (9.5)		.36	
		CORE-OM risk score	2.3 (4.0)		2.2 (3.6)		.68		1.9 (3.6)		1.8 (3.1)		.86	
		Total RRI-C-SF^c^ score	33.6 (5)		34.3 (5)		.14		33.4 (5.5)		35.8 (3.7)		.002	
		RRI-C-SF genuineness score	17.1 (2.9)		17.6 (2.5)		.057		17.1 (2.9)		18.6 (1.8)		<.001	
		RRI-C-SF realism score	16.5 (3.0)		16.7 (2.7)		.41		16.4 (3.0)		17.2 (2.5)		.06	
		Total WAI-SR^d^ score	52.1 (13)		53.0 (13)		.44		51.6 (13.5)		55.6 (11.2)		.047	
		Goal score	17.6 (4.9)		17.7 (4.9)		.90		17.5 (5.3)		18.6 (4.5)		.18	
		Task score	16.3 (4.7)		16.6 (4.7)		.43		16.0 (5.0)		17.5 (4.1)		.047	
		Bond score	18.2 (4.5)		18.7 (4.4)		.21		18.1 (4.6)		19.6 (4.2)		.04	
		GAD-7^e^	8.9 (6)		8.7 (5)		.57		8.3 (5.3)		8.2 (5.4)		.89	
		PHQ-9^f^	10.5 (6)		10.5 (6)		.97		10.5 (6.4)		9.8 (6.0)		.50	
**Psychotherapy characteristics**														
	**Treatment length (months), n (%)**							.58						.61
		0-3	47 (20.3)		37 (15.2)				14 (13.9)		5 (7.7)			
		4-6	27 (11.6)		24 (9.9)				12 (11.9)		5 (7.7)			
		7-12	27 (11.6)		32 (13.2)				13 (12.9)		9 (13.8)			
		13-24	27 (11.6)		29 (11.9)				9 (8.9)		8 (12.3)			
		>24	104 (44.8)		121 (49.8)				53 (52.5)		38 (58.5)			
	**Session frequency, n (%)**							.53						.07
		2-3 per month	128 (55.2)		127 (52.3)				51 (50.5)		23 (35.4)			
		1 per week	90 (38.8)		105 (43.2)				42 (41.6)		39 (60)			
		≥2 per week	14 (6)		11 (4.5)				8 (7.9)		3 (4.6)			
	**Treatment setting, n (%)**							.40						.35
		In person and remote mixed	9 (4)		7 (2.9)				5 (5)		0 (0)			
		In person mixed	13 (5.6)		8 (3.3)				7 (6.9)		1 (1.5)			
		Only face to face in person	67 (28.9)		67 (27.6)				27 (26.7)		20 (30.8)			
		Only in person on the couch	8 (3.4)		3 (1.2)				5 (5)		2 (3.1)			
		Only telephone call	13 (5.6)		17 (7)				4 (4)		3 (4.6)			
		Only video call	118 (50.9)		133 (54.7)				52 (51.2)		38 (58.5)			
		Remote mixed	4 (1.7)		8 (3.3)				1 (1)		1 (1.5)			
	**Treatment location, n (%)**							.08						.06
		Private practice	156 (67.2)		188 (77.4)				67 (66.3)		53 (81.5)			
		Private health institution	30 (12.9)		19 (7.8)				20 (19.8)		5 (7.7)			
		Public health institution	31 (13.4)		20 (8.2)				10 (9.9)		3 (4.6)			
		University counseling center	6 (2.6)		9 (3.7)				1 (1)		3 (4.6)			
		Other	9 (3.9)		7 (2.9)				3 (3)		1 (1.5)			
	**Session(s) canceled by patient (last 5 weeks), n (%)**							.25						.07
		No	177 (76)		196 (81)				83 (82.2)		60 (92.3)			
		Yes	55 (24)		47 (19)				18 (17.8)		5 (7.7)			
	**Disclosure with the therapist of emotional states toward them (last 5 weeks), n (%)**							.76						.11
		Never	100 (43.1)		105 (43.2)				50 (49.5)		19 (29.2)			
		Sometimes	67 (28.9)		70 (28.8)				30 (29.7)		29 (44.6)			
		About half the time	19 (8.2)		17 (7)				7 (6.9)		6 (9.2)			
		Most of the time	24 (10.3)		33 (13.6)				8 (7.9)		8 (12.3)			
		Always	22 (9.5)		18 (7.4)				6 (5.9)		3 (4.6)			
	**Therapist gender, n (%)**							.61						.14
		Woman	179 (77.2)		180 (74.1)				72 (71.3)		55 (84.6)			
		Man	48 (20.7)		59 (24.3)				27 (26.7)		9 (13.8)			
		Other	5 (2.3)		4 (1.6)				2 (2)		1 (1.5)			

^a^The N sums to more than the total sample size because cases could have more than one diagnosis.

^b^CORE-OM: Clinical Outcomes in Routine Evaluation–Outcome Measure.

^c^RRI-C-SF: Real Relationship Inventory-Client-short form.

^d^WAI-SR: Working Alliance Inventory-Short Revised.

^e^GAD-7: Generalized Anxiety Disorder-7.

^f^PHQ-9: Patient Health Questionnaire-9.

### Dropout Predictors

Logistic regression analysis revealed distinct predictors of dropout within the control group and intervention group. Analysis of the whole sample, including treatment arm and interaction terms, revealed several findings. Patients with higher baseline psychological distress (CORE-OM scores) were less likely to achieve the outcome (*β*=–0.04, *P*=.02), while those with higher baseline depressive symptoms (PHQ-9 baseline score) were more likely to achieve it (*β*=0.16, *P*=.004). Undergoing psychotherapy for more than 24 months (*β*=1.28, *P*=.009) and having a male therapist (*β*=1.07, *P*=.02) also increased the likelihood of achieving the outcome. Conversely, a history of session cancellations in the 5 weeks before the trial reduced the likelihood of achieving the outcome (*β*=–0.99, *P*=.02).

Significant interactions were found between being in the intervention group and being in a university counseling center (*β*=4.01, *P*=.02) and between being in the intervention group and having a male therapist (*β*=–2.07, *P*=.001). This means that within the intervention group, being treated in a university counseling center significantly decreased the likelihood of dropout, whereas having a male therapist increased the likelihood of dropout.

In the control group, patients who, at baseline, exhibited higher levels of psychological distress (CORE-OM baseline scores) had a reduced likelihood of completing the trial (*β*=–0.04, *P*=.02). Conversely, elevated levels of depressive symptoms (PHQ-9 baseline score; *β*=0.16, *P*=.004), undergoing psychotherapy for more than 2 years (*β*=1.28, *P*=.009), and having a male therapist notably increased the likelihood of completing the trial (*β*=1.07, *P*=.02). Additionally, a history of session cancellations in the 5 weeks preceding the trial’s onset was significantly associated with a reduced likelihood of completing the trial (*β*=–0.99, *P*=.02).

In the intervention group, patients with male therapists showed lower odds of completing the trial compared with those with female therapists (*β*=–1.00, *P*=.04).

### Per-Protocol Analyses

#### Comparative Features in the Intervention and Control Groups

At baseline, there were significant statistical differences between the intervention group (n=65) and control group (n=101) regarding 4 clinical features (Multimedia Appendix 1). All participants in the intervention group were diagnosed with a psychiatric disorder (*P*=.03) and reported an average higher level of genuineness in the real relationship with their therapists (*P*<.001) and a better quality of the task (*P*=.047) and bond (*P*=.04) dimensions of the working alliance.

#### Outcome Measures

Table 2 reports the means and standard deviations for the primary (CORE-OM) and secondary (RRI-C-SF and WAI-SR) outcomes at baseline (T0), mid-trial (T1), and the end of the trial (T2), as well as effect sizes. At baseline, the control group showed a higher, though not statistically significant, average mean score for CORE-OM (50.47 vs 47.60) and lower, though not statistically significant, average mean scores for RRI-C-SF (33.44 vs 35.85) and WAI-SR (51.58 vs 55.63).

At T2, both groups showed significant improvement in levels of psychological distress as assessed with the CORE-OM, with both reaching a total score of just over 43. The within-group effect sizes for the CORE-OM were *d*=–0.18 for the intervention group and *d*=–0.31 for the control group. ANCOVA analyses revealed no significant differences between the groups for the CORE-OM and its subscales (see Table 3). For the results shown in Table 3, ANCOVAs were conducted with respective baseline values as covariates and patients’ baseline levels of psychological distress (CORE-OM); baseline levels of anxiety (GAD-7) and depressive (PHQ-9) symptoms; baseline quality of real relationship (RRI-C-SF) and working alliance (WAI-SR); and setting, length, and session frequency of treatment as exploratory variables. When assessing the total score for each of the 3 scales, only the total score at the baseline of the respective scale was utilized as a covariate, excluding the subscores. Conversely, in the analysis of each subscale score, all subscores (excluding the total score) at baseline of the relevant scale serve as covariates. The Cohen *d* calculation uses adjusted means from ANCOVA models. Paired sample *t* tests indicated a significant decrease in psychological distress from T0 to T2 for both the intervention group (total score: t_64_=2.38, *P*=.02; functioning subscore: t_64_=2.53, *P*=.01) and control group (total score: t_100_=3.84, *P*<.001; well-being subscore: t_100_=3.20, *P*=.002; symptoms subscore: t_100_=4.08, *P*<.001; functioning subscore: t_100_=2.84, *P*=.006; risk subscore: t_100_=2.08, *P*=.04; see Table 4).

**Table 2:** Within-group analyses for primary and secondary outcomes for the per-protocol sample.

Measurements	Control group							Intervention group					
	T0 (n=101), mean (SD)	T1 (n=101), mean (SD)	T2 (n=101), mean (SD)	T0-T1, Cohen *d* (95% CI)	T1-T2, Cohen *d* (95% CI)	T0-T2, Cohen *d* (95% CI)	T0 (n=65), mean (SD)		T1 (n=65), mean (SD)	T2 (n=65), mean (SD)	T0-T1, Cohen *d* (95% CI)	T1-T2, Cohen *d* (95% CI)	T0-T2, Cohen *d* (95% CI)
CORE-OM^a^	50.47 (24.50)	44.05 (21.95)	43.14 (22.06)	–0.28 (–0.55 to 0.00)	–0.04 (–0.32 to 0.23)	–0.31 (–0.59 to –0.04)	47.60 (24.46)		46.17 (25.29)	43.32 (23.92)	–0.06 (–0.33 to –0.22)	–0.12 (–0.39 to –0.16)	–0.18 (–0.45 to –0.10)
CORE-OM well-being	7.68 (3.71)	6.88 (3.65)	6.68 (3.42)	–0.22 (–0.49 to –0.06)	–0.06 (–0.33 to 0.22)	–0.28 (–0.56 to 0.00)	7.52 (3.96)		7.35 (4.08)	6.92 (4.01)	–0.04 (–0.32 to 0.23)	–0.11 (–0.38 to 0.17)	–0.15 (–0.43 to 0.13)
CORE-OM symptoms	21.83 (10.79)	18.81 (9.60)	18.15 (9.62)	–0.30 (–0.57 to –0.02)	–0.07 (–0.35 to 0.21)	–0.36 (–0.64 to –0.08)	20.57 (9.97)		20.17 (11.05)	19.09 (10.12)	–0.04 (–0.31 to 0.24)	–0.10 (–0.38 to 0.17)	–0.15 (–0.42 to 0.13)
CORE-OM functioning	19.01 (9.15)	17.09 (8.78)	16.93 (8.99)	–0.21 (–0.49 to 0.06)	–0.02 (–0.29 to 0.26)	–0.23 (–0.51 to –0.05)	17.66 (9.54)		16.86 (9.54)	15.75(9.40)	–0.08 (–0.36 to 0.19)	–0.12 (–0.39 to 0.16)	–0.20 (–0.48 to 0.07)
CORE-OM risk	1.94 (3.60)	1.27 (2.38)	1.38 (2.69)	–0.22 (–0.50 to 0.06)	0.04 (–0.23 to 0.32)	–0.18 (–0.45 to 0.10)	1.85 (3.13)		1.79 (2.86)	1.55 (2.56)	–0.02 (–0.30 to 0.26)	–0.09 (–0.36 to 0.19)	–0.11 (–0.38 to 0.17)
RRI-C-SF^b^	33.44 (5.47)	34.09 (4.86)	33.84 (5.57)	0.13 (–0.15 to 0.40)	–0.05 (–0.32 to 0.23)	0.07 (–0.20 to 0.35)	35.85 (3.74)		35.39 (4.02)	35.29 (4.00)	–0.12 (–0.40 to 0.16)	–0.02 (–0.30 to 0.25)	–0.15 (–0.42 to 0.13)
RRI-C-SF genuineness	17.05 (2.91)	17.41 (2.59)	17.09 (2.98)	0.13 (–0.15 to 0.41)	–0.12 (–0.39 to 0.16)	0.01 (–0.26 to 0.28)	18.62 (1.79)		18.11 (1.92)	18.11 (1.94)	–0.28 (–0.55 to 0.00)	0.00 (–0.28 to 0.28)	–0.27 (–0.55 to 0.00)
RRI-C-SF realism	16.39 (2.99)	16.68 (2.63)	16.75 (2.89)	0.10 (–0.17 to 0.38)	0.03 (–0.25 to 0.30)	0.12 (–0.15 to 0.40)	17.23 (2.47)		17.28 (2.51)	17.19 (2.55)	0.02 (–0.26 to 0.30)	–0.04 (–0.31 to 0.24)	–0.02 (–0.29 to 0.26)
WAI-SR^c^	51.58 (13.53)	54.26 (13.18)	53.81 (13.92)	0.12 (–0.08 to 0.33)	–0.04 (–0.28 to 0.20)	0.08 (–0.14 to 0.30)	55.63 (11.25)		55.69 (12.97)	55.79 (12.19)	0.00 (–0.27 to 0.28)	0.01(–0.27 to 0.28)	0.01 (–0.26 to 0.29)
WAI-SR goal	17.50 (5.31)	18.43 (5.04)	18.14 (5.19)	0.20 (–0.08 to 0.48)	–0.03 (–0.31 to 0.24)	0.16 (–0.11 to 0.44)	18.59 (4.55)		18.59 (5.11)	18.71 (4.87)	0.00 (–0.28 to 0.28)	0.02 (–0.25 to 0.30)	0.03 (–0.25 to 0.30)
WAI-SR task	15.98 (4.96)	17.07 (4.85)	17.10 (5.09)	0.22 (–0.05 to 0.50)	0.01 (–0.27 to 0.28)	0.22 (–0.05 to 0.50)	17.46 (4.14)		17.20 (4.77)	17.31 (4.82)	–0.06 (–0.33 to 0.22)	0.02 (–0.25 to 0.30)	–0.03 (–0.31 to 0.24)
WAI-SR bond	18.11 (4.55)	18.76 (4.50)	18.57 (4.61)	0.14 (–0.13 to 0.42)	–0.04 (–0.32 to 0.23)	0.10 (–0.18 to 0.38)	19.59 (4.16)		19.91 (4.07)	19.77 (3.82)	0.08 (–0.20 to 0.35)	–0.04 (–0.31 to 0.24)	0.05 (–0.23 to 0.32)

^a^CORE-OM: Clinical Outcomes in Routine Evaluation–Outcome Measure.

^b^RRI-C-SF: Real Relationship Inventory-Client-short form.

^c^WAI-SR: Working Alliance Inventory-Short Revised.

**Table 3:** Between-group comparisons for primary and secondary outcomes.

Outcomes	T1				T2		
	*F* (*df*)	*P* value	Cohen *d* (95% CI)	*F* (*df*)		*P* value	Cohen *d* (95% CI)
CORE-OM^a^	0.80 (1,d2)	.37	–0.16 (–0.47 to 0.15)	0.01 (1,d2)		.94	–0.15 (–0.46 to 0.16)
CORE-OM well-being	1.36 (1,d2)	.25	–0.10 (–0.42 to 0.21)	0.32 (1,d2)		.57	–0.13 (–0.45 to 0.18)
CORE-OM symptoms	1.47 (1,d2)	.23	–0.17 (–0.48 to 0.15)	0.73 (1,d2)		.40	–0.25 (–0.57 to 0.06)
CORE-OM functioning	0.06 (1,d2)	.80	–0.08 (–0.39 to 0.23)	1.37 (1,d2)		.24	–0.05 (–0.37 to 0.26)
CORE-OM risk	2.78 (1,d2)	.10	–0.27 (–0.58 to 0.04)	0.31 (1,d2)		.58	–0.05 (–0.36 to 0.26)
RRI-C-SF^b^	7.29 (1,d2)	.008	0.00 (–0.31 to 0.31)	6.27 (1,d2)		.01	0.06 (–0.25 to 0.37)
RRI-C-SF genuineness	6.92 (1,d2)	.009	–0.09 (–0.23 to 0.40)	9.82 (1,d2)		.002	0.00 (–0.31 to 0.31)
RRI-C-SF realism	4.43 (1,d2)	.04	0.00 (–0.31 to 0.31)	1.92 (1,d2)		.17	0.07 (–0.24 to 0.38)
WAI-SR^c^	1.50 (1,d2)	.22	0.17 (–0.14 to 0.48)	1.90 (1,d2)		.17	0.13 (–0.18 to 0.44)
WAI-SR goal	0.13 (1,d2)	.72	0.16 (–0.15 to 0.47)	1.05 (1,d2)		.31	0.12 (–0.19 to 0.43)
WAI-SR task	0.07 (1,d2)	.79	0.23 (–0.08 to 0.54)	0.16 (1,d2)		.69	0.24 (–0.07 to 0.55)
WAI-SR bond	7.12 (1,d2)	.009	0.00 (–0.31 to 0.31)	5.13 (1,d2)		.03	0.02 (–0.29 to 0.33)

^a^CORE-OM: Clinical Outcomes in Routine Evaluation–Outcome Measure.

^b^RRI-C-SF: Real Relationship Inventory-Client-short form.

^c^WAI-SR: Working Alliance Inventory-Short Revised.

Regarding the secondary outcomes, the within-group effect sizes at T2 for the intervention group were small for levels of real relationship (*d*=–0.15), as assessed with the RRI-C-SF, and near zero for the quality of the working alliance (*d*=0.01), as assessed with the WAI-SR. For the control group, the within-group effect sizes were small for both the level of real relationships (*d*=0.07) and the quality of the working alliance (*d*=0.08). At T2, the intervention group still exhibited better scores for both the RRI-C-SF (35.29 vs 33.84) and WAI-SR (55.79 vs 53.81). ANCOVAs revealed statistically significant differences between the groups at T2 for the genuineness dimension of the RRI-C-SF (*F*_(1,d2)_=9.82, *P*=.002) and for the bond dimension of the WAI-SR (*F*_(1,d2)_=5.13, *P*=.03). Paired sample *t* tests showed a significant decrease in scores on the RRI-C-SF genuineness scale for the intervention group (t_64_=2.52, *P*=.01) and a significant increase in the WAI-SR total score (t_100_=–2.05, *P*=.04) and its bond subscore (t_100_=–2.93, *P*=.004) for the control group.

**Table 4:** Within-group comparisons for primary and secondary outcomes for the per-protocol sample.

Group comparisons		T0-T1				T1-T2				T0-T2		
		*t* (*df*)	*P* value	*r*	*t* (*df*)		*P* value	*r*	*t* (*df*)		*P* value	*r*
**Control group**												
	CORE-OM^a^	3.75 (100)	<.001	0.69	0.59 (100)		.55	0.78	3.84 (100)		<.001	0.70
	CORE-OM well-being	2.88 (100)	.005	0.71	0.84 (100)		.41	0.77	3.20 (100)		.002	0.61
	CORE-OM symptoms	3.61 (100)	<.001	0.67	0.92 (100)		.36	0.71	4.08 (100)		<.001	0.61
	CORE-OM functioning	2.94 (100)	.004	0.73	0.24 (100)		.81	0.73	2.84 (100)		.006	0.67
	CORE-OM risk	2.20 (100)	.03	0.53	–0.44 (100)		.66	0.53	2.08 (100)		.04	0.66
	RRI-C-SF^b^	–1.69 (100)	.09	0.81	0.65 (100)		.52	0.76	–0.86 (100)		.39	0.70
	RRI-C-SF genuineness	–1.67 (100)	.10	0.70	1.48 (100)		.14	0.71	–0.15 (100)		.88	0.59
	RRI-C-SF realism	–1.28 (100)	.20	0.66	–0.31 (100)		.76	0.68	–1.47 (100)		.15	0.64
	WAI-SR^c^	–3.26 (100)	.002	0.86	0.48 (100)		.63	0.80	–2.05 (100)		.04	0.74
	WAI-SR goal	–3.21 (100)	.002	0.84	0.78 (100)		.44	0.74	–1.55 (100)		.12	0.69
	WAI-SR task	–3.35 (100)	.001	0.78	–0.09 (100)		.93	0.77	–2.93 (100)		.004	0.71
	WAI-SR bond	–1.93 (100)	.057	0.72	0.56 (100)		.58	0.72	–1.09 (100)		.28	0.56
**Intervention group**												
	CORE-OM	0.69 (64)	.49	0.82	1.79 (64)		.08	0.77	2.38 (64)		.02	0.72
	CORE-OM well-being	0.47 (64)	.64	0.74	1.44 (64)		.16	0.82	1.79 (64)		.08	0.77
	CORE-OM symptoms	0.43 (64)	.67	0.75	1.49 (64)		.14	0.85	1.89 (64)		.06	0.80
	CORE-OM functioning	1.02 (64)	.31	0.78	1.66 (64)		.10	0.84	2.53 (64)		.01	0.79
	CORE-OM risk	0.22 (64)	.83	0.72	0.81 (64)		.42	0.65	0.95 (64)		.35	0.64
	RRI-C-SF	1.47 (64)	.15	0.64	0.31 (64)		.76	0.64	1.56 (64)		.12	0.61
	RRI-C-SF genuineness	3.09 (64)	.003	0.75	0.00 (64)		≥.99	0.75	2.52 (64)		.01	0.62
	RRI-C-SF realism	–0.20 (64)	.84	0.72	0.42 (64)		.68	0.75	0.20 (64)		.85	0.71
	WAI-SR	–0.09 (64)	.93	0.72	–0.11 (64)		.91	0.63	–0.19 (64)		.85	0.59
	WAI-SR goal	0.00 (64)	≥.99	0.85	–0.32 (64)		.75	0.81	–0.32 (64)		.75	0.78
	WAI-SR task	0.71 (64)	.48	0.79	–0.29 (64)		.77	0.81	0.47 (64)		.64	0.83
	WAI-SR bond	–1.22 (64)	.23	0.87	0.45 (64)		.65	0.81	–0.55 (64)		.56	0.77

^a^CORE-OM: Clinical Outcomes in Routine Evaluation–Outcome Measure.

^b^RRI-C-SF: Real Relationship Inventory-Client-short form.

^c^WAI-SR: Working Alliance Inventory-Short Revised.

### Intention-To-Treat Analysis

The results of the intention-to-treat analyses are provided in the appendixes. More specifically, Multimedia Appendix 2 presents the means and standard deviations for the primary and secondary outcomes at T0, T1, and T2, along with within-group Cohen *d* values. Multimedia Appendix 3 offers between-group comparisons for the primary and secondary outcomes analyzed at T1 and T2, using analyses of covariance (*F*) and the corresponding Cohen *d* values. Finally, Multimedia Appendix 4 details within-group comparisons for the primary and secondary outcomes using *t* tests and *r* correlation values for the T0-T1, T1-T2, and T0-T2 periods.

## Discussion

### Principal Findings

The limited efficacy of psychotherapeutic interventions affects about 30% of patients [32]. To address this issue, our study examined the potential of internet-based, therapist-independent interventions that encourage patient self-assessment and thus self-reflection on a crucial element of the therapeutic relationship: the emotional reactions toward the therapist during individual psychotherapy sessions. We aimed to determine if the use of this web-based routine process monitoring and feedback system over a period of 10 weeks could significantly reduce psychological distress or improve the real relationship and working alliance quality, compared with a treatment-as-usual group receiving only individual therapy.

In our per-protocol analyses, no significant differences were found across groups for the primary outcome of psychological distress, as assessed by the CORE-OM. Despite significant improvements observed over time in both groups, the difference in improvement was more notable in the control group for the primary outcome (*d*=–31 versus *d*=–18). However, it is crucial to remember that the control group had higher baseline scores (50.47 vs 47.60), which allowed more room for improvement. By the end of the trial, CORE-OM scores were nearly indistinguishable between the groups. Regarding the secondary outcomes, our findings were contrary to our initial hypothesis. The intervention group, which focused on self-assessment of in-session emotional reactions over the 10-week intervention period, did not demonstrate improvements in working alliance quality or real relationship levels. In fact, there was a decrease in the levels of real relationship observed. Compared with the control group, the intervention group had lower effect sizes concerning the quality of these therapeutic relationship elements, which were also noted with lower baseline scores in the control group.

Of particular interest was the change over time in the levels of real relationship between the groups. The most significant difference was observed in the genuineness dimension, with the intervention group showing an effect size of *d*=–0.27, contrasted with *d*=0.01 in the control group at T2. Although this finding is not consistent with our original hypotheses (ie, an increase in both genuineness and realism scores), it provides an important clinical insight, as well as a potentially important theoretical insight. Our post hoc interpretation relies on the fact that the items composing the genuineness subscale of the RRI-C-SF relate to being oneself with the therapist, openness and honesty, the ability to communicate one’s moment-to-moment inner experience, and the expression of feelings in therapy. Thus, it is possible to hypothesize that the intervention—which required patients to reflect on the aforementioned specific aspects of their personal relationship with their therapist—may have helped the patients to gain a greater awareness of certain inner struggles or resistances of him/her/them within the relationship. In this sense, the lower scores observed in the RRI-C-SG genuineness dimension do not signify a worsening of the real relationship with the therapist; instead, they might denote a more realistic and accurate perception of it. The maintenance of the bond dimension of the working alliance (*d*=0.05), corroborates the nondeterioration of the relationship. Furthermore, at the end of trial, the effect sizes for the task dimension of the working alliance were *d*=0.22 for the control group and *d*=–0.03 for the intervention group, allowing us to hypothesize that a focus on the emotional aspects of the therapeutic relationship might reduce the emphasis on or perceived importance of therapy tasks.

Another important finding is the very high attrition rate observed in the intervention group, where 45% of the patients who completed the postsession battery at least once eventually dropped out. This substantial dropout rate suggests that the implementation of this particular form of routine self-monitoring may not be feasible or acceptable for many patients. Notably, in the intervention group (but not in the control group), patients who dropped out had statistically significantly lower levels of real relationship (*P*=.002), lower quality of the working alliance (*P*=.051), and less disclosure of their emotional states to the therapist (*P*=.01) compared with patients who did not drop out. This might imply that patients who perceived the quality of their therapeutic relationship as subpar were less motivated to engage with it. Furthermore, since our intervention included encouraging patients to discuss their in-session emotional reactions with their therapist, it is important to recognize that evidence indicates that disclosure in psychotherapy is positively correlated with the strength of the therapeutic alliance [33] and that common psychotherapy-related deceptions often involve patients feigning agreement with the therapist’s comments [34]—and thus concealment of real emotional reactions toward such comments. Therefore, patients experiencing a lower quality of alliance may have been more likely to withdraw from a study that required them to engage in behaviors, like direct emotional disclosure, that they are unaccustomed to.

Taken together, our findings on retention and outcomes only partially support the notion that a systematic monitoring process and feedback system, involving only the patient, is a viable and effective addition to individual psychotherapy. However, future research should test our hypothesis that the statistically significant lower scores observed in the genuineness dimension of the real relationship might indicate a more realistic and accurate perception by the patients. If this proves true, utilizing our postsession battery over a longer period could lead to a significantly more accurate self-perception of their levels of genuineness with the therapist. This might be clinically significant, given the direct relationship between the quality of the real relationship and session and treatment outcomes [35], as well as its close association with the working alliance [36], which in turn is closely linked to treatment outcomes [37,38]. Additionally, future research should explore the use of this battery as a tool administered within psychotherapy sessions—at the very start, referring to the previous session—so that therapists and patients can discuss it directly. This method of monitoring process or outcome and feedback to the therapist is currently the most widely used [13,16].

Some of the findings were post hoc and unexpected. It is possible that asking about the sessions and affect raised the salience of these issues. The study design did not provide scores nor feedback to the therapist. This may have brought feelings to the surface while not providing a context to process them in the next session. The larger plan for a “feedback system” is intended to include such a mechanism, and the role of “salience without processing” is a testable hypothesis that should be addressed in future work. Possibilities include focus groups or “exit interviews” with participants, as well as development of an integrated monitoring and reporting system.

From a practical perspective, if future research confirms the hypothesis that our online self-monitoring system fosters a more realistic and accurate perception of the therapeutic relationship from the patient’s point of view, it could have significant clinical and economic benefits. In this case, the system should be adapted to include the therapist, who would receive the patient’s self-assessed outcome and use this information to guide the psychotherapeutic intervention. This could strengthen the therapeutic relationship and improve treatment outcomes. Although this monitoring and feedback system would involve the participation of the therapist, it would not increase their assessment workload. Therapists would only need to review the feedback and determine how to incorporate it into the therapeutic process. This system could be applied in various therapeutic settings without imposing additional costs on patients or excessive demands on therapists, making it highly cost efficient. By allowing more frequent and systematic monitoring of patient progress without requiring in-person assessments, such tools could reduce both the duration and overall cost of treatment. In fact, research shows that feedback systems not only improve outcomes but also lower costs associated with prolonged therapy and high attrition rates [18]. These economic advantages are especially relevant in resource-limited health care settings, where optimizing the efficiency of therapeutic interventions is essential to expand access to care [18,39].

### Limitations

Due to the nature of online recruitment and assessment, all data collected in this study were self-reported. Although we recommended that participants discuss the study intervention with their therapists, it is unclear whether they followed this advice or what the therapists’ reactions were (whether encouraging, discouraging, or neutral). Given that some therapists may be skeptical about research, some of them might not have actively encouraged participation (or might even have discouraged it), potentially adversely affecting the study’s attrition rate. Another limitation concerns the types of psychotherapeutic approaches used (eg, psychodynamic, cognitive behavioral). We did not collect information on the specific type of psychotherapy used, which could influence the results. Last, participation in the study did not require specific demographic nor clinical characteristics, such as particular diagnoses or levels of psychological distress. This approach enabled the inclusion of a broad spectrum of individuals undergoing individual psychotherapy. Although this heterogeneity expanded the reach of our study and reflected a realistic clinical setting, it also introduced a level of variability that may affect the generalizability and interpretation of our findings.

### Conclusion

Although routine monitoring and feedback systems involving therapists have consistently demonstrated effectiveness in various trials, the benefits of internet-based systems that solely involve the patient remain uncertain. In this study, our new web-based intervention aimed at enhancing self-monitoring and reflection on emotional responses did not yield the expected benefits and appears to be somewhat disadvantageous in terms of the quality of the therapeutic relationship. However, we identified a notable finding: Our postsession battery significantly influences the patients’ perception of the genuineness dimension of the real relationship. This outcome suggests a promising avenue for future research and potential clinical applications, underscoring the need for further investigation.

## Appendix

Multimedia Appendix 1 Differences in demographics, clinical, and treatment characteristics between patients who dropped out and those who did not.

Multimedia Appendix 2 Within-group analyses for primary and secondary outcomes for the intention-to-treat sample.

Multimedia Appendix 3 Between-group comparisons for primary and secondary outcomes for the intention-to-treat sample.

Multimedia Appendix 4 Within-group comparisons for primary and secondary outcomes for the intention-to-treat sample.

Multimedia Appendix 5 CONSORT eHEALTH checklist (V 1.6.1).

## Abbreviations

ANCOVA: analyses of covariance
CORE-OM: Clinical Outcomes in Routine Evaluation–Outcome Measure
GAD-7: Generalized Anxiety Disorder-7
PHQ-9: Patient Health Questionnaire-9
RISQ: Rift In-Session Questionnaire
RRI-C-SF: Real Relationship Inventory-Client-short form
SPARQ: in-Session Patient Affective Reactions Questionnaire
WAI-SR: Working Alliance Inventory-Short Revised
