# Supplementary material for: Effectiveness of a Novel Web-Based Intervention to Enhance Therapeutic Relationships and Treatment Outcomes in Adult Individual Psychotherapy: Randomized Controlled Trial and Analysis of Predictors of Dropouts
Source: JMIR Ment Health. 2024 Nov 27;11:e63234. doi: 10.2196/63234 (PMC11635334; doi:10.2196/63234)
Supplement: Multimedia Appendix 1 [file mental_v11i1e63234_app1.docx]

**Table S1.** Differences in demographics, clinical, and treatment characteristics between patients who dropped out and those who did not.

|  | **Control group** | |  | **Intervention group** | |  |
| --- | --- | --- | --- | --- | --- | --- |
|  | Dropouts ^a^ | Per-Protocol |  | Dropouts | Per-Protocol |  |
|  | (*n* = 131) | (*n* = 101) | *p* | (*n* = 178) | (*n* = 65) | *p* |
| *Sociodemographic features* |  |  |  |  |  |  |
| Age (years) |  |  | 0.796 |  |  | 0.231 |
| 18-22 | 6 (4.6%) | 8 (7.9%) |  | 14 (7.9%) | 2 (3.1%) |  |
| 23-29 | 38 (29.0%) | 23 (22.8%) |  | 45 (25.3%) | 9 (13.8%) |  |
| 30-39 | 41 (31.3%) | 33 (32.7%) |  | 49 (27.5%) | 19 (29.2%) |  |
| 40-49 | 18 (13.7%) | 12 (11.9%) |  | 31 (17.4%) | 15 (23.1%) |  |
| 50-59 | 17 (13.0%) | 15 (14.9%) |  | 19 (10.7%) | 10 (15.4%) |  |
| ≥60 | 11 (8.4%) | 10 (9.9%) |  | 20 (11.2%) | 10 (15.4%) |  |
| Gender |  |  | 0.656 |  |  | 0.437 |
| Woman | 103 (78.6%) | 73 (72.3%) |  | 131 (73.6%) | 54 (83.1%) |  |
| Man | 18 (13.7%) | 20 (19.8%) |  | 30 (16.9%) | 8 (12.3%) |  |
| Other | 9 (6.9%) | 7 (6.9%) |  | 16 (9.0%) | 3 (4.6%) |  |
| Prefer not to say | 1 (0.8%) | 1 (1.0%) |  | 1 (0.6%) | 0 (0.0%) |  |
| Education |  |  | 0.227 |  |  | 0.685 |
| Less than high school | 3 (2.3%) | 0 (0.0%) |  | 0 (0.0%) | 0 (0.0%) |  |
| High school graduate | 10 (7.6%) | 5 (5.0%) |  | 5 (2.8%) | 1 (1.5%) |  |
| Some college | 30 (22.9%) | 14 (13.9%) |  | 30 (16.9%) | 9 (13.8%) |  |
| 2 year degree | 12 (9.2%) | 10 (9.9%) |  | 17 (9.6%) | 2 (3.1%) |  |
| 4 year degree | 35 (26.7%) | 38 (37.6%) |  | 66 (37.1%) | 21 (32.3%) |  |
| Professional degree | 35 (26.7%) | 27 (26.7%) |  | 50 (28.1%) | 25 (38.5%) |  |
| Doctorate | 6 (4.6%) | 7 (6.9%) |  | 10 (5.6%) | 7 (10.8%) |  |
| *Clinical characteristics* |  |  |  |  |  |  |
| Diagnosis of a mental disorder |  |  | 0.097 |  |  | **0.002** |
| No | 18 (13.7%) | 7 (6.9%) |  | 23 (12.9%) | 0 (0.0%) |  |
| Yes | 113 (86.3%) | 94 (93.1%) |  | 155 (87.1%) | 65 (100.0%) |  |
| Diagnoses |  |  |  |  |  |  |
| Anxiety disorders | 92 | 79 | 0.320 | 132 | 53 | **<0.001** |
| Bipolar disorders | 24 | 11 | **0.028** | 25 | 12 | **0.033** |
| Depressive disorders | 74 | 68 | 0.615 | 106 | 49 | **<0.001** |
| Disruptive behavior and dissocial disorders | 3 | 0 | 0.083 | 1 | 3 | 0.317 |
| Eating disorders | 15 | 14 | 0.853 | 16 | 5 | **0.016** |
| Neurodevelopmental disorders | 37 | 31 | 0.467 | 48 | 17 | **<0.001** |
| Post-traumatic stress disorder | 51 | 46 | 0.612 | 54 | 28 | **0.004** |
| Psychotic disorders | 2 | 3 | 0.655 | 4 | 1 | 0.180 |
| Cluster A personality disorder | 2 | 1 | 0.564 | 4 | 0 | **0.046** |
| Cluster B personality disorder | 14 | 12 | 0.695 | 11 | 8 | 0.491 |
| Cluster C personality disorder | 17 | 5 | **0.011** | 8 | 5 | 0.405 |
| Other(s) | 6 | 5 | 0.763 | 16 | 5 | **0.016** |
| Psychiatric medication use |  |  | 0.138 |  |  | 0.305 |
| No | 47 (35.9%) | 27 (26.7%) |  | 50 (28.1%) | 14 (21.5%) |  |
| Yes | 84 (64.1%) | 74 (73.3%) |  | 128 (71.9%) | 51 (78.5%) |  |
| Medication stability |  |  | 0.686 |  |  | 0.588 |
| 1 month or less | 9 (10.7%) | 8 (10.8%) |  | 16 (12.5%) | 6 (11.8%) |  |
| 2 months | 13 (15.5%) | 8 (10.8%) |  | 11 (8.6%) | 7 (13.7%) |  |
| 3 months or more | 62 (73.8%) | 58 (78.4%) |  | 101 (78.9%) | 38 (74.5%) |  |
| Psychological measures |  |  |  |  |  |  |
| CORE-OM total score (mean*, SD*) | 55.2 (23.7) | 50.5 (24.5) | 0.139 | 53.1 (23.1) | 47.6 (24.5) | 0.104 |
| Wellbeing score (mean*, SD*) | 8.3 (3.6) | 7.7 (3.7) | 0.197 | 8.1 (3.5) | 7.5 (4.0) | 0.217 |
| Symptoms score (mean*, SD*) | 23.0 (10.8) | 21.8 (10.8) | 0.419 | 22.8 (10.3) | 20.6 (10.0) | 0.130 |
| Functioning score (mean*, SD*) | 21.3 (9.0) | 19.0 (9.1) | 0.056 | 19.9 (9.0) | 17.7 (9.5) | 0.096 |
| Risk score (mean*, SD*) | 2.6 (4.2) | 1.9 (3.6) | 0.218 | 2.3 (3.7) | 1.8 (3.1) | 0.400 |
| RRI-C-SF total score (mean*, SD*) | 33.7 (5.4) | 33.4 (5.5) | 0.673 | 33.73 (4.9) | 35.8 (3.7) | **0.002** |
| Genuineness score (mean*, SD*) | 17.1 (2.9) | 17.1 (2.9) | 0.821 | 17.2 (2.6) | 18.6 (1.8) | **<0.001** |
| Realism score (mean*, SD*) | 16.6 (3.0) | 16.4 (3.0) | 0.584 | 16.5 (2.7) | 17.2 (2.5) | 0.070 |
| WAI-SR total score (mean*, SD*) | 52.4 (12.1) | 51.6 (13.5) | 0.621 | 52.0 (13.3) | 55.6 (11.2) | **0.051** |
| Goal score (mean*, SD*) | 17.7 (4.6) | 17.5 (5.3) | 0.759 | 17.3 (5.0) | 18.6 (4.5) | 0.079 |
| Task score (mean*, SD*) | 16.5 (4.4) | 16.0 (5.0) | 0.405 | 16.3 (4.9) | 17.5 (4.1) | 0.090 |
| Bond score (mean*, SD*) | 18.2 (4.4) | 18.1 (4.6) | 0.840 | 18.4 (4.5) | 19.6 (4.2) | 0.059 |
| GAD-7 (mean*, SD*) | 9.4 (5.7) | 8.3 (5.3) | 0.117 | 8.8 (5.3) | 8.2 (5.4) | 0.386 |
| PHQ-9 (mean*, SD*) | 10.5 (6.3) | 10.5 (6.4) | 0.970 | 10.7 (5.9) | 9.8 (6.0) | 0.290 |
| *Psychotherapy characteristics* |  |  |  |  |  |  |
| Treatment length (months) |  |  | 0.119 |  |  | 0.276 |
| 0 to 3 | 33 (25.2%) | 14 (13.9%) |  | 32 (18.0%) | 5 (7.7%) |  |
| 4 to 6 | 15 (11.5%) | 12 (11.9%) |  | 19 (10.7%) | 5 (7.7%) |  |
| 7 to 12 | 14 (10.7%) | 13 (12.9%) |  | 23 (12.9%) | 9 (13.8%) |  |
| 13 to 24 | 18 (13.7%) | 9 (8.9%) |  | 21 (11.8%) | 8 (12.3%) |  |
| more than 24 | 51 (38.9%) | 53 (52.5%) |  | 83 (46.6%) | 38 (58.5%) |  |
| Session frequency |  |  | 0.346 |  |  | **0.015** |
| 2 to 3 per month | 77 (58.8%) | 51 (50.5%) |  | 104 (58.4%) | 23 (35.4%) |  |
| 1 per week | 48 (36.6%) | 42 (41.6%) |  | 66 (37.1%) | 39 (60.0%) |  |
| 2 or more per week | 6 (4.6%) | 8 (7.9%) |  | 8 (4.5%) | 3 (4.6%) |  |
| Treatment setting |  |  | 0.679 |  |  | 0.244 |
| in person and remote mixed | 4 (3.1%) | 5 (5.0%) |  | 7 (3.9%) | 0 (0.0%) |  |
| in person mixed | 6 (4.6%) | 7 (6.9%) |  | 7 (3.9%) | 1 (1.5%) |  |
| only in person face to face | 40 (30.5%) | 27 (26.7%) |  | 47 (26.4%) | 20 (30.8%) |  |
| only in person on the couch | 3 (2.3%) | 5 (5.0%) |  | 1 (0.6%) | 2 (3.1%) |  |
| only telephone call | 9 (6.9%) | 4 (4.0%) |  | 14 (7.9%) | 3 (4.6%) |  |
| only video call | 66 (50.4%) | 52 (51.5%) |  | 95 (53.4%) | 38 (58.5%) |  |
| remote mixed | 3 (2.3%) | 1 (1.0%) |  | 7 (3.9%) | 1 (1.5%) |  |
| Treatment location |  |  | **0.036** |  |  | 0.669 |
| Private practice | 89 (67.9%) | 67 (66.3%) |  | 135 (75.8%) | 53 (81.5%) |  |
| Private health institution | 10 (7.6%) | 20 (19.8%) |  | 14 (7.9%) | 5 (7.7%) |  |
| Public health institution | 21 (16.0%) | 10 (9.9%) |  | 17 (9.6%) | 3 (4.6%) |  |
| University counseling center | 5 (3.8%) | 1 (1.0%) |  | 6 (3.4%) | 3 (4.6%) |  |
| Other | 6 (4.6%) | 3 (3.0%) |  | 6 (3.4%) | 1 (1.5%) |  |
| Session(s) cancelled by patient (last 5 weeks) |  |  | 0.064 |  |  | **0.005** |
| No | 94 (71.8%) | 83 (82.2%) |  | 136 (76.4%) | 60 (92.3%) |  |
| Yes | 37 (28.2%) | 18 (17.8%) |  | 42 (23.6%) | 5 (7.7%) |  |
| Disclosure with the therapist of emotional states toward them (last 5 weeks) |  |  | 0.242 |  |  | **0.010** |
| Never | 50 (38.2%) | 50 (49.5%) |  | 86 (48.3%) | 19 (29.2%) |  |
| Sometimes | 37 (28.2%) | 30 (29.7%) |  | 41 (23.0%) | 29 (44.6%) |  |
| About half the time | 12 (9.2%) | 7 (6.9%) |  | 11 (6.2%) | 6 (9.2%) |  |
| Most of the time | 16 (12.2%) | 8 (7.9%) |  | 25 (14.0%) | 8 (12.3%) |  |
| Always | 16 (12.2%) | 6 (5.9%) |  | 15 (8.4%) | 3 (4.6%) |  |
| Therapist gender |  |  | 0.137 |  |  | 0.210 |
| Woman | 107 (81.7%) | 72 (71.3%) |  | 125 (70.2%) | 55 (84.6%) |  |
| Man | 21 (16.0%) | 27 (26.7%) |  | 50 (28.1%) | 9 (13.8%) |  |
| Other | 3 (2.3%) | 2 (2.0%) |  | 3 (1.7%) | 1 (1.5%) |  |

^a^ This category also includes patients who did not complete T1 but did complete T2.
