# Supplementary material for: Effectiveness of a Novel Web-Based Intervention to Enhance Therapeutic Relationships and Treatment Outcomes in Adult Individual Psychotherapy: Randomized Controlled Trial and Analysis of Predictors of Dropouts
Source: JMIR Ment Health. 2024 Nov 27;11:e63234. doi: 10.2196/63234 (PMC11635334; doi:10.2196/63234)
Supplement: Multimedia Appendix 2 [file mental_v11i1e63234_app2.docx]

**Table S2.** Within-group analyses for primary and secondary outcomes for the intention-to-treat sample.

|  | Control group | | | | | |  | Intervention group | | | | | |
| --- | --- | --- | --- | --- | --- | --- | --- | --- | --- | --- | --- | --- | --- |
| Measurements | T0  (*n*=232)  Mean (*SD*) | T1  (*n*=149)  Mean (*SD*) | T2  (*n*=125)  Mean (*SD*) | Cohen’s *d*  (95% CI)  T0-T1 | Cohen’s *d*  (95% CI)  T1-T2 | Cohen’s *d*  (95% CI)  T0-T2 |  | T0  (*n*=243)  Mean (*SD*) | T1  (*n*=125)  Mean (*SD*) | T2  (*n*=65)  Mean (*SD*) | Cohen’s *d*  (95% CI)  T0-T1 | Cohen’s *d*  (95% CI)  T1-T2 | Cohen’s *d*  (95% CI)  T0-T2 |
| CORE-OM | 53.13  (24.10) | 46.07  (23.58) | 43.84  (23.15) | –0.30  (–0.50, –0.09) | –0.10  (–0.33, –0.14) | –0.39  (–0.61, 0.17) |  | 51.66  (23.54) | 45.57  (24.74) | 43.32  (23.92) | –0.25  (–0.47, –0.04) | –0.09  (–0.39, –0.21) | –0.35  (–0.63, –0.08) |
| Wellbeing | 8.03  (3.63) | 7.13  (3.76) | 6.76  (3.56) | –0.24  (–0.45, –0.04) | –0.10  (–0.34, 0.14) | –0.35  (–0.57, 0.13) |  | 8.00  (3.63) | 7.10  (3.80) | 6.92  (4.01) | –0.24  (–0.46, –0.03) | –0.05  (–0.35, –0.25) | –0.29  (–0.57, –0.02) |
| Symptoms | 22.49 (10.81) | 19.64 (10.30) | 18.54  (9.96) | –0.27  (–0.48, –0.06) | –0.11  (–0.35, –0.13) | –0.38  (–0.60, –0.16) |  | 22.21 (10.21) | 19.67 (11.16) | 19.09 (10.12) | –0.24  (–0.46, –0.02) | –0.05  (–0.35, –0.25) | –0.31  (–0.58, –0.03) |
| Functioning | 20.31  (9.09) | 17.94  (9.38) | 17.05  (9.35) | –0.26  (–0.46, –0.05) | –0.10  (–0.33, –0.14) | –0.35  (–0.57, –0.14) |  | 19.29  (9.20) | 17.20  (9.37) | 15.75  (9.40) | –0.23  (–0.44, –0.01) | –0.16  (–0.46, –0.15) | –0.38  (–0.66, –0.11) |
| Risk | 2.31  (3.96) | 1.36  (2.65) | 1.50  (2.77) | –0.27  (–0.48, –0.06) | –0.05  (–0.19, 0.29) | –0.23  (–0.44, –0.01) |  | 2.17  (3.56) | 1.59  (3.10) | 1.55  (2.56) | –0.17  (–0.39, –0.05) | –0.01  (–0.31, –0.29) | –0.18  (–0.46, –0.09) |
| RRI-C-SF | 33.61  (5.43) | 33.73  (5.41) | 34.02  (5.46) | 0.02  (–0.18, 0.23) | –0.06  (–0.29, 0.18) | 0.08  (–0.14, 0.29) |  | 34.29  (4.72) | 35.02  (3.93) | 35.29  (4.00) | 0.16  (–0.05, 0.38) | 0.07  (–0.23, 0.37) | 0.22  (–0.06, 0.49) |
| Genuineness | 17.10  (2.92) | 17.20  (2.85) | 17.24  (2.92) | 0.03  (–0.17, 0.24) | 0.01  (–0.22, 0.25) | 0.01  (–0.20, 0.23) |  | 17.57  (2.47) | 17.89  (2.03) | 18.11  (1.94) | 0.14  (–0.08, 0.35) | 0.11  (–0.19, 0.41) | 0.23  (–0.05, 0.50) |
| Realism | 16.51  (2.98) | 16.53  (2.91) | 16.79  (2.89) | 0.01  (–0.20, 0.21) | 0.09  (–0.15, 0.33) | 0.10  (–0.12, 0.31) |  | 16.72  (2.66) | 17.13  (2.40) | 17.19  (2.55) | 0.16  (–0.06, 0.38) | 0.02  (–0.28, 0.32) | 0.17  (–0.10, 0.45) |
| WAI-SR | 52.06  (12.72) | 53.66  (13.25) | 53.12  (14.15) | 0.12  (–0.08, 0.33) | –0.04  (–0.28, 0.20) | 0.08  (–0.14, 0.30) |  | 52.97  (12.84) | 55.56  (12.73) | 55.79  (12.19) | 0.20  (–0.01, 0.42) | 0.02  (–0.28, 0.32) | 0.22  (–0.05, 0.50) |
| Goal | 17.61  (4.91) | 18.19  (5.12) | 17.84  (5.42) | 0.12  (–0.09, 0.32) | –0.07  (–0.30, 0.17) | 0.05  (–0.17, 0.26) |  | 17.66  (4.94) | 18.48  (4.94) | 18.71  (4.87) | 0.17  (–0.05, 0.38) | 0.05  (–0.25, 0.35) | 0.21  (–0.06, 0.49) |
| Task | 16.27  (4.67) | 16.70  (4.95) | 16.82  (5.11) | 0.09  (–0.12, 0.30) | 0.02  (–0.21, 0.26) | 0.11  (–0.10, 0.33) |  | 16.61  (4.71) | 17.33  (4.82) | 17.31  (4.82) | 0.15  (–0.06, 0.37) | –0.00  (–0.30, 0.30) | 0.15  (–0.13, 0.42) |
| Bond | 18.18  (4.47) | 18.78  (4.46) | 18.46  (4.67) | 0.13  (–0.07, 0.34) | –0.07  (–0.31, 0.17) | 0.06  (–0.16, 0.28) |  | 18.70  (4.44) | 19.75  (4.09) | 19.77  (3.82) | 0.24  (0.03, 0.50) | 0.01  (–0.29, 0.31) | 0.25  (–0.02, 0.53) |
