# Supplementary material for: Effectiveness of a Novel Web-Based Intervention to Enhance Therapeutic Relationships and Treatment Outcomes in Adult Individual Psychotherapy: Randomized Controlled Trial and Analysis of Predictors of Dropouts
Source: JMIR Ment Health. 2024 Nov 27;11:e63234. doi: 10.2196/63234 (PMC11635334; doi:10.2196/63234)
Supplement: Multimedia Appendix 3 [file mental_v11i1e63234_app3.docx]

**Table S3.** Between-group comparisons for primary and secondary outcomes for the intention-to-treat sample.

|  | T1 |  |  |  | T2 |  |  |
| --- | --- | --- | --- | --- | --- | --- | --- |
|  | *F* | *p* | Cohen’s *d* |  | *F* | *p* | Cohen’s *d* |
| CORE-OM | 1.25 | 0.260 | –0.09  (–0.33, 0.14) |  | 0.02 | 0.887 | –0.10  (–0.40, 0.20) |
| Wellbeing | 1.09 | 0298 | –0.02  (–0.26, 0.22) |  | 0.00 | 0.949 | –0.09  (–0.39, 0.21) |
| Symptoms | 0.26 | 0.610 | –0.14  (–0.37, 0.10) |  | 0.17 | 0.681 | –0.12  (–0.42, 0.18) |
| Functioning | 3.59 | 0.059 | –0.04  (–0.28, 0.19) |  | 0.48 | 0.487 | –0.07  (–0.37, 0.23) |
| Risk | 0.08 | 0.773 | –0.18  (–0.42, 0.06) |  | 0.34 | 0.560 | –0.05  (–0.35, 0.25) |
| RRI-C-SF | 8.30 | **0.004** | –0.02  (–0.26, 0.22) |  | 5.87 | **0.016** | 0.04  (–0.26, 0.34) |
| Genuineness | 6.67 | **0.010** | –0.04  (–0.28, 0.19) |  | 6.34 | **0.013** | 0.00  (–0.30, 0.30) |
| Realism | 5.45 | **0.020** | –0.04  (–0.28, 0.20) |  | 3.23 | 0.074 | 0.07  (–0.23, 0.37) |
| WAI-SR | 1.43 | 0.233 | 0.06  (–0.18, 0.30) |  | 2.65 | 0.105 | 0.06  (–0.24, 0.36) |
| Goal | 0.00 | 0.990 | 0.10  (–0.14, 0.34) |  | 1.27 | 0.261 | 0.04  (–0.14, 0.22) |
| Task | 0.93 | 0.336 | 0.06  (–0.18, 0.30) |  | 0.85 | 0.358 | 0.14  (–0.16, 0.44) |
| Bond | 4.94 | **0.027** | –0.02  (–0.26, 0.21) |  | 5.84 | **0.016** | 0.00  (–0.30, 0.30) |

*Note.* Analysis of covariances with respective baseline values as covariates and patient’s baseline levels of psychological distress (CORE-OM), baseline levels of anxiety (GAD-7) and depressive (PHQ-9) symptoms, baseline quality of real relationship (RRI-C-SF) and working alliance (WAI-SR), as well as setting, length, and session frequency of treatment, as exploratory variables. When assessing the total score for each of the three scales, only the total score at the baseline of the respective scale is utilized as a covariate, excluding the subscores. Conversely, in the analysis of each subscale score, all subscores (excluding the total score) at baseline of the relevant scale serve as covariates. The Cohen *d* calculation uses adjusted means from ANCOVA models.
