# Supplementary material for: Effectiveness of a Novel Web-Based Intervention to Enhance Therapeutic Relationships and Treatment Outcomes in Adult Individual Psychotherapy: Randomized Controlled Trial and Analysis of Predictors of Dropouts
Source: JMIR Ment Health. 2024 Nov 27;11:e63234. doi: 10.2196/63234 (PMC11635334; doi:10.2196/63234)
Supplement: Multimedia Appendix 4 [file mental_v11i1e63234_app4.docx]

**Supplementary Table 4**

Within-group comparisons for primary and secondary outcomes for the intention-to-treat sample.

|  |  | T0-T1 |  |  |  | T1-T2 |  |  |  | T0-T2 |  |  |
| --- | --- | --- | --- | --- | --- | --- | --- | --- | --- | --- | --- | --- |
|  |  | *t (df)* | *p* | *r* |  | *t (df)* | *p* | *r* |  | *t (df)* | *p* | *r* |
| Control group |  |  |  |  |  |  |  |  |  |  |  |  |
| CORE-OM |  | 4.80 (148) | **<0.001** | 0.76 |  | 0.59 (100) | 0.554 | 0.75 |  | 4.17 (124) | **<0.001** | 0.68 |
| Wellbeing |  | 3.56 | **<0.001** | 0.72 |  | 0.84 | 0.406 | 0.77 |  | 3.69 | **<0.001** | 0.60 |
| Symptoms |  | 4.60 | **<0.001** | 0.71 |  | 0.92 | 0.362 | 0.71 |  | 3.94 | **<0.001** | 0.60 |
| Functioning |  | 3.61 | **<0.001** | 0.75 |  | 0.24 | 0.809 | 0.73 |  | 3.56 | **<0.001** | 0.69 |
| Risk |  | 3.38 | **<0.001** | 0.63 |  | –0.44 | 0.660 | 0.53 |  | 2.03 | **0.044** | 0.66 |
| RRI-C-SF |  | –0.34 | 0.731 | 0.76 |  | 0.65 | 0.519 | 0.74 |  | –1.33 | 0.191 | 0.66 |
| Genuineness |  | –0.48 | 0.629 | 0.71 |  | 1.48 | 0.141 | 0.71 |  | –0.40 | 0.694 | 0.63 |
| Realism |  | –0.11 | 0.913 | 0.71 |  | –0.31 | 0.755 | 0.68 |  | –2.03 | **0.045** | 0.67 |
| WAI-SR |  | –2.57 | **0.011** | 0.80 |  | 0.48 | 0.630 | 0.77 |  | –1.79 | 0.076 | 0.67 |
| Goal |  | –2.37 | **0.019** | 0.80 |  | 0.78 | 0.435 | 0.74 |  | –0.94 | 0.351 | 0.66 |
| Task |  | –2.06 | **0.042** | 0.73 |  | –0.09 | 0.930 | 0.77 |  | –2.33 | **0.022** | 0.69 |
| Bond |  | –2.01 | **0.047** | 0.75 |  | 0.56 | 0.578 | 0.72 |  | –1.58 | 0.116 | 0.58 |
| Intervention group |  |  |  |  |  |  |  |  |  |  |  |  |
| CORE-OM |  | 2.21 (124) | **0.029** | 0.73 |  | 1.79 (64) | 0.079 | 0.79 |  | 2.38 (64) | **0.021** | 0.73 |
| Wellbeing |  | 1.63 | 0.105 | 0.67 |  | 1.44 | 0.155 | 0.70 |  | 1.79 | 0.079 | 0.69 |
| Symptoms |  | 2.14 | **0.034** | 0.66 |  | 1.49 | 0.142 | 0.76 |  | 1.89 | 0.063 | 0.69 |
| Functioning |  | 1.83 | 0.069 | 0.76 |  | 1.66 | 0.101 | 0.79 |  | 2.53 | **0.014** | 0.70 |
| Risk |  | 1.14 | 0.255 | 0.56 |  | 0.81 | 0.421 | 0.53 |  | 0.95 | 0.347 | 0.68 |
| RRI-C-SF |  | 0.45 | 0.655 | 0.75 |  | 0.31 | 0.760 | 0.69 |  | 1.56 | 0.124 | 0.66 |
| Genuineness |  | 1.87 | 0.063 | 0.71 |  | 0.00 | 1.000 | 0.64 |  | 2.52 | **0.014** | 0.59 |
| Realism |  | –0.81 | 0.417 | 0.67 |  | 0.42 | 0.679 | 0.65 |  | 0.20 | 0.845 | 0.62 |
| WAI-SR |  | –1.61 | 0.111 | 0.84 |  | –0.11 | 0.914 | 0.72 |  | –0.19 | 0.853 | 0.70 |
| Goal |  | –0.94 | 0.348 | 0.81 |  | –0.32 | 0.748 | 0.74 |  | –0.32 | 0.751 | 0.66 |
| Task |  | –0.91 | 0.365 | 0.79 |  | –0.29 | 0.772 | 0.68 |  | 0.47 | 0.643 | 0.72 |
| Bond |  | –2.30 | **0.023** | 0.80 |  | 0.45 | 0.653 | 0.64 |  | –0.55 | 0.585 | 0.67 |
